# Enhancing Three-Dimensional Reconstruction Through Intelligent Colormap Selection

**DOI:** 10.3390/s25082576

**Published:** 2025-04-18

**Authors:** Alexandros Vrochidis, Dimitrios Tzovaras, Stelios Krinidis

**Affiliations:** 1 Department of Management Science and Technology, Democritus University of Thrace, 65404 Kavala, Greece; krinidis@mst.duth.gr; 2 Center for Research and Technology Hellas, Information Technologies Institute, 57001 Thessaloniki, Greece; dimitrios.tzovaras@iti.gr

**Keywords:** structure-from-motion, 3D reconstruction, keypoint detection, colormap optimization, underwater reconstruction

## Abstract

Photogrammetry is the process of creating three-dimensional (3D) reconstructions from two-dimensional images. In this paper, a novel method is introduced that significantly enhances 3D reconstruction by improving image quality through a combination of RGB stretching, Contrast Limited Adaptive Histogram Equalization (CLAHE), and colormaps. This approach effectively increases the number of keypoints and matches between images, resulting in more accurate and detailed 3D reconstructions. Additionally, a heuristic methodology is proposed to identify the optimal colormaps for each dataset based on keypoint matches between image pairs. This approach saves valuable time by recommending the most effective colormaps, eliminating the need to test each individually. A new dataset comprising two real-world image collections from underwater cultural heritage sites is presented to validate the algorithm, characterized by challenging environments such as low visibility and varying lighting conditions. The limitations of existing techniques are addressed by this method, providing a robust solution for enhancing image quality in demanding underwater scenarios. Experimental results show that image enhancement can lead to a 7.91% and 11.4% improvement, and the enhancement fusion with colormaps improves reconstruction accuracy by 10.82% and 64.11%. These findings render the proposed methodology a promising tool for 3D reconstruction in challenging environments, like underwater.

## 1. Introduction

Structure-from-Motion (SfM) is a photogrammetric technique that reconstructs 3D structures from a series of 2D images taken from different viewpoints. It is crucial in fields such as computer vision, where understanding the geometry of an environment is essential for tasks like navigation and object recognition. It plays a crucial role in various fields, including archaeology, where it helps preserve cultural heritage [1], human digitization [2], and virtual reality experiences [3]. Modern techniques leverage advancements in Structure-from-Motion (SfM) and Multi-View Stereo (MVS) to achieve high accuracy and detail. In [4], a comprehensive analysis and refinement of the traditional SfM techniques was presented, and a methodology named COLMAP was introduced. The methodology addressed common challenges in SfM, such as handling large-scale datasets and improving robustness in diverse and complex scenes. Another popular methodology is OpenSfM [5], a solution designed with scalability and modularity, offering flexible integration with other services, making it popular for projects that prioritize extensibility and adaptability. SFM methodologies often face challenges when processing degraded images. To address these limitations, approaches such as [6,7] focus on improving image illumination and enhancing visual quality, ensuring that the images retain more meaningful information for further analysis.

In this paper, a novel methodology to enhance 3D reconstruction by fusing pre-processing techniques such as RGB stretching and CLAHE with colormaps is proposed. By utilizing colormaps, more features can be identified, leading to better image stitching and more accurate 3D reconstructions. The proposed enhancement methodology showed a rise of 7.91% and 11.4%, while colormaps increased the features by 10.82% and 64.11% for the first and second experimental datasets, respectively. Additionally, a heuristic approach for selecting the optimal colormaps for each dataset is introduced. Although various methodologies exist for enhancing 3D reconstruction, none combine colormaps with color enhancement to address the issue of light absorption, a common challenge in underwater environments. The main innovations presented in this paper include:

- A novel methodology that combines colormaps and color enhancement methods to improve 3D reconstruction;
- A heuristic approach identifying the optimal colormaps for each dataset;
- A fusion of preprocessing techniques that enhance 3D reconstruction;
- An evaluation of 3D reconstruction and the effects of colormaps and enhancement techniques using real-world datasets.

Initially, the dataset images are enhanced using a fusion of CLAHE and RGB stretching. These enhanced images are then processed by a developed methodology that selects two random images, applies 22 colormaps, and counts the number of feature matches between them. The colormap with the fewest matches is eliminated, and the process continues with two new random images and the remaining colormaps until the optimal colormap is identified. Once the best colormap for the dataset is determined, the enhanced images are transformed using this colormap and processed with OpenSfM for 3D reconstruction. Importantly, the final reconstruction step utilizes the original, unprocessed images, ensuring that while image matches are maintained, the resulting 3D model retains its original colors, which is crucial.

In contrast to conventional SfM methodologies, which are robust but struggle with low-contrast or light-absorbing environments, this approach introduces a unique fusion of RGB stretching and CLAHE with an array of colormaps. This fusion enhances subtle image features, typically lost in challenging conditions, such as underwater scenes. By systematically evaluating 22 different colormaps and applying a heuristic selection process, the method adapts the enhancement to each specific dataset, ensuring the optimal balance between feature richness and computational efficiency. Moreover, processing the final 3D reconstruction using the original images preserves the authentic color information, which is critical for applications in cultural heritage, human digitization, and virtual reality. These enhancements collectively address key limitations of current techniques and offer a flexible, scalable, and more accurate solution for 3D reconstruction in diverse imaging conditions.

The remainder of this paper is as follows: Section 2 provides a concise overview of related work. Section 3 outlines the details of the proposed 3D reconstruction framework. Section 4 discusses the methodology evaluation, including the relevant metrics used. Finally, Section 5 presents the conclusions of the study, along with its limitations and suggestions for future improvements.

## 2. Related Work

A novel indoor scene reconstruction method, introduced in [8], is based on the Manhattan-world assumption. The core concept involves using semantic information from planar regions to aid in geometry reconstruction. The method learns 3D semantics from 2D segmentation results and then jointly optimizes them with the geometry, improving robustness against inaccurate 2D segmentation. In [9], a novel method for the reconstruction and completion of 3D shapes from sparse point clouds was introduced. It first uses a neural network to learn the best transformation for the data and then applies a regression technique to fit the transformed data points into a complete 3D shape. In [10], a CNN-based method was proposed for multi-view 3D reconstruction using transformers. Unlike previous CNN methods, this method unifies the feature extraction and view fusion in a single transformer network, reducing the parameters by 70% and increasing accuracy.

In [11], a real-time coherent 3D reconstruction method from monocular video was proposed. The key idea is the joint reconstruction and fusion of the 3D geometry directly in the volumetric truncated signed distance function representation. In [12], a deep implicit moving least-squares surface technique for 3D reconstruction was presented, which can handle various data point sets and utilize the approximation power of implicit surface representations. Its efficacy and generalization ability were well demonstrated through extensive tests. In [13], the issue of poor image registration caused by visual patterns, which occur more times in one scene, was addressed. The proposed method identified a neighborhood of reliable images using a similarity measure and utilized these images to calculate initial pose parameters. This approach reduces runtime, applies to any incremental SfM system, and enhances reconstruction for various challenging scenes.

In [14], a system to reconstruct stylized 3D character heads from illustrated anime character portraits was proposed. These characters have hair with more complex and dispersed geometry, making their reconstruction more demanding. Their method outperformed baseline methods and provided data to establish the task of stylized reconstruction from portrait illustrations. Another method was proposed in [15] for 3D reconstruction of dressed humans from sparse views. The data-driven end-to-end approach reconstructed an implicit 3D representation of dressed humans from sparse camera views. In [16], fiducial markers were utilized to enhance image matching and resectioning order and improve reconstruction time and accuracy. The methodology was developed on top of OpenSfM. In [17], a method for reducing the OpenSfM time by 30% and increasing the reconstructed points by approximately 25% was developed. This improvement was achieved by integrating local 3D environment models using camera trajectories corresponding to image sequences with overlapped images. A similar technique was proposed in [18], where an image selection method was described to improve the computational efficiency of 3D reconstruction. A threshold was set for the optical flow of the image sequence, ensuring that only images meeting this criterion were used. A remotely operated robot obtained these images.

In [19], a 3D reconstruction of an underwater environment was proposed using 2D sonar images. To avoid noise and low resolution, researchers utilized sonar imaging. They employed a Convolutional Neural Network (CNN) to analyze incoming images in real-time, selectively using a subset for feature annotation. In [20], sonar technology was also employed for the surface reconstruction of an inspected structure. The proposed algorithm employs BlueView imaging and operates in near real-time, paving the way for further online 3D applications. A drawback of these methods is that employing sonar images may result in information loss compared to high-quality underwater imagery, which offers richer details about texture. This problem can be solved using SfM technologies that can utilize images to reconstruct a 3D environment even under poor light conditions.

In [21], a LiDAR imaging sensor was used for real-time 3D scene reconstruction using a GPU for real-time image reconstruction. The system used a 532 nm pulsed laser source and measured photon time-of-flight with picosecond resolution. Three-dimensional imaging was achieved for targets up to 7.5 attenuation lengths away, with real-time video processing at 10 frames per second. LiDAR can be fused with multibeam sonar, enhancing the results [22]. The method involves adaptive multi-step processing, including noise reduction, object detection, and point cloud triangulation for surface reconstruction. Various shape reconstruction algorithms were tested, highlighting their strengths and weaknesses. LiDAR technology can achieve less than 1% error [23]. This study presented an automated system using computer vision and optical sensors to create a precise 3D model of an elevator shaft without human input. The setup includes a Jetson Nano, LiDAR, laser sensors, and an MPU, which capture the shaft’s smallest cross-section in a single pass. Validation experiments showed high accuracy, enabling reliable measurements.

Photogrammetry plays a crucial role in the documentation and preservation of Underwater Cultural Heritage (UCH) sites [24]. This review discussed the challenges of underwater documentation and explored the use of optical sensors and photogrammetric techniques for producing high-quality, textured 3D models. Key aspects of the underwater 3D reconstruction process, such as data acquisition, color restoration, and advanced techniques like Structure-from-Motion (SfM) [25] and Multi-View Stereo (MVS) [26], were analyzed for their effectiveness in creating accurate models. Photogrammetry was emphasized as the most widely used non-invasive and cost-effective method for generating high-resolution 3D models and 2D orthomosaics underwater [27]. Recent applications were reviewed, underscoring ongoing challenges and requirements in UCH documentation.

Recent research has shown the effectiveness of photogrammetry in documenting and preserving cultural heritage by generating precise 3D models of historical structures [28]. SfM has been combined with deep learning to improve tunnel inspections, overcoming inefficiencies and high costs in traditional methods [29]. This approach utilizes a Mask R-CNN for defect segmentation and incorporates the identified defects into a high-quality 3D tunnel model created from images taken every 1 m. Successfully applied in a Shanghai metro tunnel, the SfM-deep learning method proved reliable for enhancing intelligent tunnel detection by visualizing and quantifying leakage defects. A fusion of photogrammetry with unmanned aerial vehicles can enhance the 3D reconstruction and provide accurate results with less than 1 cm error [30]. They were used to create a hyper-realistic 3D model of a campus, utilizing images and high-accuracy GPS data. The project highlights optimized UAV techniques for historical preservation, facility maintenance, campus planning, and potential virtual reality (VR) applications.

The comparison presented in Table 1 highlights several key characteristics of related works in the field of 3D reconstruction, emphasizing aspects like real-world applicability, cost-effectiveness, scalability, support for multiple datasets, and performance in poor lighting conditions. Some of these methods are effective in controlled conditions, but their scalability and ability to work in diverse real-world scenarios are limited, especially when dealing with complex environments or poor lighting. Methods that require sensors like sonar demonstrate robust performance in challenging underwater environments. However, their high cost and specialized equipment limit their broader applicability and scalability. On the other hand, photogrammetry techniques offer cost-effective solutions that scale well across multiple datasets and environments. However, their effectiveness under poor lighting remains a challenge.

The proposed method distinguishes itself by introducing a novel and scalable approach that performs well under diverse real-world conditions and addresses the challenges of poor lighting environments. The methodology leverages RGB stretching, CLAHE, and colormaps to optimize feature matching and improve the overall reconstruction accuracy. This innovative approach is especially valuable in low-light environments, where traditional methods often fail to deliver high-quality results, making it a critical advancement for scenarios such as underwater reconstructions.

## 3. Enhanced 3D Reconstruction Through Intelligent Colormap Selection

### 3.1. Image Enhancement

The first step involved enhancing the image colors. After evaluating four different enhancement methods and their combinations, it was found that the fusion of RGB stretching [17] together with CLAHE [18] significantly improved feature matching and surpassed the others. RGB stretching was applied by expanding the histograms of the red, green, and blue channels, thereby increasing image contrast and distributing pixel intensity levels. This technique is particularly beneficial for underwater scenarios, where sunlight penetration differs greatly from terrestrial environments. For instance, red light is absorbed within the first 5 m, orange at 10 m, yellow at 20 m, green at 30 m, and blue at 60 m. By applying RGB stretching, these color absorption challenges are addressed, ensuring better image clarity and color representation. The formula used for RGB stretching is presented below:

(1) $$P_{out}=\left(P_{in\;}-L_{min}\right)\left(\frac{V_{max}-V_{min}}{L_{max}-L_{min}}\right)+V_{min}$$

where *P_in_* and *P_out_* are the input and output pixels, respectively. *L_max_* and *L_min_* represent the maximum and minimum intensity level values for the input image, and *V_min_* and *V_max_* denote the intensity values for the output images.

The other method used to enhance the image contrast is the CLAHE. It is a method employed in image processing to enhance the local contrast and improve the image quality. Unlike traditional histogram equalization methods, CLAHE divides the image into smaller regions named tiles and then performs histogram equalization on each one. Dividing the image into tiles allows for adaptive adjustments based on local contrast characteristics. The image noise is reduced by limiting the contrast enhancement in each tile. The approach ensures that bright and dark areas receive enhancement and have a better visual appearance. It is particularly effective in scenarios of poor lighting conditions, which is the case in underwater environments. It is also effective in images containing a wide range of intensity levels. It is a valuable tool in various fields, including medical imaging and underwater photography.

**Figure 1:**
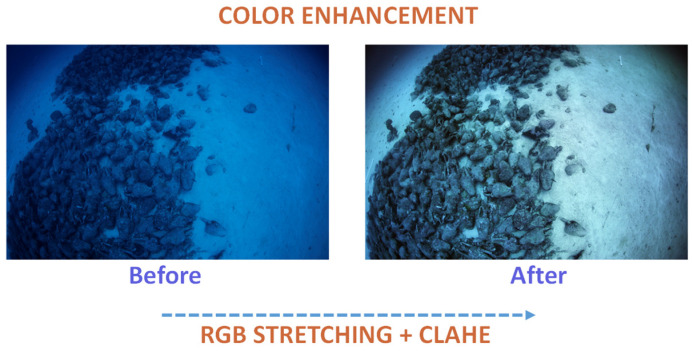
The color enhancement visualization.

The fusion of the two techniques starts by stretching the images’ RGB channels and then applying the CLAHE method. The result is an image with better quality that allows the detection of more features. An indicative example of the aforementioned color enhancement fusion is visualized in Figure 1. The figure features an image from a real-world underwater dataset. The image depicts the amphorae of Peristera, located at a depth of 30 m near Skopelos. After the fusion of CLAHE and RGB stretching, the application of colormaps follows, and its formula is presented below:

(2) $$\;I_{colormap\;}(x,y)=C\left(I_{enhanced\;}(x,y)\right)$$

where *I_colormap_* (*x*,*y*) is the image coming from the colormap application after the fusion of CLAHE and RGB stretching. *x* and *y* represent the width and height values, while *I_enhanced_* is the enhanced image, and *C*(.) is the colormap function that maps grayscale values to colors.

It is important to note that first, the image turns to grayscale, and then the colormap is applied. This method ensures that the colormap enhances the intensity variations meaningfully. Colormaps map grayscale intensity values to colors using predefined schemes. For instance, the HSV colormap does not perform a color space conversion but instead assigns colors to intensity values based on the HSV color space. The result is a smooth transition through hues, providing a visually appealing representation of the grayscale data.

### 3.2. Efficient Colormap Selection Using Heuristics

After enhancing the dataset images with preprocessing techniques, a heuristic algorithm was employed to find the optimal colormaps. This systematic method iteratively evaluates and refines the set of candidate colormaps based on their performance. By progressively narrowing down the colormap set and eliminating the worst-performing ones at each iteration, the heuristic effectively guides the search toward an optimal solution, which is the best colormap for the dataset in this case. Heuristic techniques are used in optimization problems where finding the optimal solution is impractical or impossible within a reasonable time. Instead, heuristics aim to efficiently find good solutions, even if they may not guarantee the best solution.

The heuristic methodology begins by randomly selecting an image from the dataset and then choosing its sequential image. This approach reduces the risk of failing to find matches, especially when the dataset comes from sequential video frames. If they are not sequential, it will still function by identifying fewer matches. For the initial pair of images, 22 colormaps [31] were applied, and the number of matches for each colormap was counted. The colormaps used were autumn, bone, jet, winter, rainbow, ocean, summer, spring, cool, hsv, pink, hot, parula, magma, inferno, plasma, viridis, cividis, twilight, twilight_shifted, turbo, and deepgreen. These colormaps were chosen to provide a varied yet concise selection that balances the number of possibilities with the need for effective presentation. This collection ensured ease of use and simplicity. While reducing the number of examined colormaps may impact stitching accuracy, it also lowers computational costs. Performance and efficiency have to be balanced in the limited options.

Initially, the colormap with the worst performance, based on image matches, was removed. The remaining colormaps were stored in a list. Next, a new image was selected randomly, along with its sequential image. These images were processed with the remaining colormaps, and the results were added to the existing results to update the overall classification. The colormap with the worst performance was removed again. This process was repeated until the best colormap was identified. The process is presented in Algorithm 1. The initial evaluation was based on the formula below:
**Algorithm 1:** Heuristic Approach for Finding the Optimal Colormaps**Data:** Images, cmaps_list, cmaps_to_reduce01:    Set total_results to an empty list02:    for n in range(len(cmaps_list), 0, -cmaps_to_reduce):03:            Randomly select an image and its sequential pair from the dataset04:            Enhance the images using a fusion of RGB stretching and CLAHE05:             Apply each colormap from cmaps_list to the images and calculate the number of keypoint matches, storing results in colormaps_df06:            Append the results from colormaps_df to total_results07:            Sort total_results to identify the best colormaps08:            Remove the worst-performing colormap from cmaps_list09:            Write intermediate results to a text file10:    **return** The best colormaps from the sorted total_results

(3) $$\left\{{C^{\prime }}_{1},\;{C^{\prime }}_{2},\ldots ,{C^{\prime }}_{n}\right\}={Top}_{n}\left(\left\{E(I,C_{i})|C_{i}\in C\right\}\right)$$

where the *Top_n_* (.) function selects the top *n* colormaps based on the evaluation metric *Ε*, which is the match number between two consecutive images. *C* represents the colormaps, and *C_i_* denotes the *i*-th colormap from the available colormaps set. *I* denotes the input image, and is *E* the evaluation metric, which quantifies the quality of the resulting image after applying the colormap *C* to image *I*. Then the iterative selection follows the formula below:

(4) $$C_{best}={argmax}_{C\prime \in \left\{{C^{\prime }}_{1},\;{C^{\prime }}_{2},\ldots ,{C^{\prime }}_{21}\right\}}E(I,C^{\prime })$$

where *C_best_* denotes the best colormap and argmax is a function that returns the top *n* colormaps that were given as input. For each iteration, the top colormaps follow the (2) and continue the iterative process until the top-asked colormaps are found.

**Figure 2:**
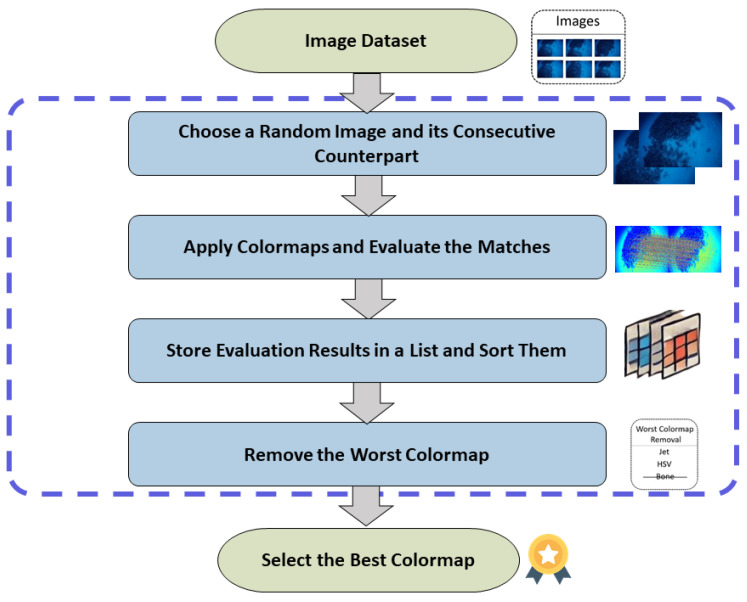
Flow chart of the proposed heuristic methodology.

The flowchart of the heuristic algorithm, shown in Figure 2, begins with an input image dataset. It then randomly selects an image and its consecutive counterpart, applying various colormaps and counting the matches between them for each colormap. The evaluation results are stored in a list, which is then sorted. The algorithm iteratively removes the worst-performing colormap until the best colormap is identified. Finally, the entire image dataset is processed using this optimal colormap.

### 3.3. Enhancing 3D Reconstruction with Colormaps

Once the best colormap was identified in terms of keypoint matches between frames, it transformed the images, which were then given as input to the 3D reconstruction. The methodology for 3D reconstruction was based on OpenSfM and began with extracting metadata from the images. This metadata included image dimensions and, if available, GPS coordinates, capture time, projection type, and focal ratio. The next step was detecting image features, which were improved using colormaps and the proposed color-enhancing method. After feature detection, matches between images were identified. The methodology first determined the list of image pairs to analyze and then performed matching detection for each pair to find corresponding feature points. This process can be time-consuming due to the large number of possible image pairs, but the presence of GPS coordinates or capture time can significantly reduce the time required.

**Figure 3:**
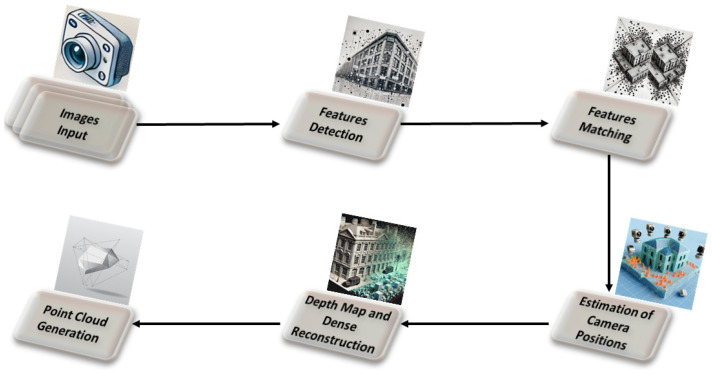
The architectural framework of the SfM methodology illustrating the key computational steps and processes involved.

Once matches between image pairs were identified, the matches were linked to build feature track points. A track is a collection of feature points from different images identified as corresponding to the same physical point. The reconstruction process then began, aiming to determine the 3D positions of the tracks along with the camera positions, which represent the motion. Next, triangular meshes were added to the reconstruction to enable smooth transitions between viewers. Corrected versions of the images were created, removing lens distortions and preparing the data for depth map computation. In the next step, a detailed 3D point cloud was generated from the undistorted images and reconstructions. The final result of this methodology was the point cloud. This process utilized enhanced images only for keypoint detection to improve matching, while the unprocessed images were used to create the final point cloud. The procedure is illustrated in Figure 3.

**Figure 4:**
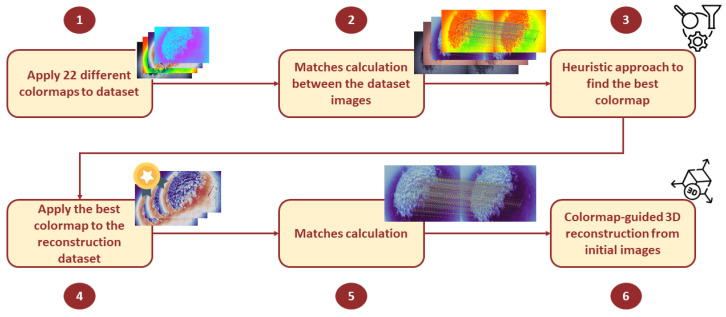
The architecture of the proposed methodology.

After enhancing the image, the process involved applying a specific colormap to the dataset images. This step is crucial as it enables the keypoint detection methodology to identify more keypoints, leading to better matches between images. After applying the colormap matches, calculations started, and reconstruction followed. It is important to note that the reconstruction should be based on the original images, not the colormap-applied ones. The colormap is only used to increase the keypoint matches. Reconstructing the initial images increases the number of matches and enhances the quality of the 3D reconstruction. This improvement occurs because the dataset remains the same, but the images are better stitched together. The whole process of heuristic finding the most appropriate colormaps, together with the reconstruction, is presented in Figure 4.

While colormaps are utilized in image matching, they play a crucial role in the proposed method for generating the initial point cloud by significantly enhancing feature extraction and overall reconstruction accuracy. It is important to emphasize that colormaps are used solely for image matching. The overall reconstruction is performed using the initial images, which retain all the original details without incorporating colormaps. This contribution goes beyond preprocessing, addressing key limitations in existing methods, as demonstrated by the experimental results presented in the paper.

### 3.4. Implementation Details

The Scale-Invariant Feature Transform (SIFT) [32] was used to detect keypoints. In addition to SIFT, other methods such as Speeded Up Robust Features (SURF) [33], Accelerated KAZE (AKAZE) [34], Hessian Affine feature point detector combined with HOG descriptor (HAHOG) [35], and Oriented FAST and Rotated BRIEF (ORB) [36] were also tested. SIFT was chosen due to its balanced combination of accuracy and efficiency. The scale independence of SIFT is highly advantageous for the heuristic algorithm, as it allows images to be downscaled, thereby reducing computational costs and increasing processing speed without compromising the accuracy. However, any other keypoint detection method can be used with the proposed methodology. The CLAHE hyperparameters were tuned with a tile size of (4, 4) and clip 2.

The experiments were implemented with no problems using four threads. OpenSfM is primarily CPU-based, with its core Structure-from-Motion optimization relying on the Ceres Solver, which does not support GPU acceleration. Additionally, the depth map estimation techniques are also CPU-intensive and do not require or utilize GPU hardware for processing. Therefore, OpenSfM does not have GPU acceleration needs and operates fully on the CPU. All experiments were conducted on a system equipped with an Intel i5-10600K CPU, 16 GB of RAM, and an Nvidia RTX 1660 GPU. The equipment used in the experiments was sourced from the United States. Specifically, the system included components manufactured by Intel Corporation and NVIDIA Corporation, both headquartered in Santa Clara, California, USA.

## 4. Experimental Evaluation

Experiments were conducted to assess the effectiveness of various colormaps and their impact on 3D reconstruction. Initially, a heuristic methodology was employed to validate whether colormaps identified as optimal truly exhibited superior performance. Subsequently, reconstructions were performed using 22 different colormaps. The experimental evaluation utilized real-world datasets to ensure assessment under realistic conditions. This Section presents a detailed account of these experiments along with the corresponding results.

### 4.1. Dataset

The first dataset (dataset A) consisted of 280 underwater images captured near Skopelos at a depth of 30 m. These images of both datasets depict the amphorae of Peristera, originating from a shipwreck dated to the last quarter of the 5th century BC based on typological characteristics. The site features a dense concentration of amphorae, forming a low hill measuring 25 m in length and 12 m in width, and contains approximately 1000 amphorae. The total number of load amphorae was estimated at 4200, which is the largest in size and capacity of the classical period that has been discovered to date.

**Figure 5:**
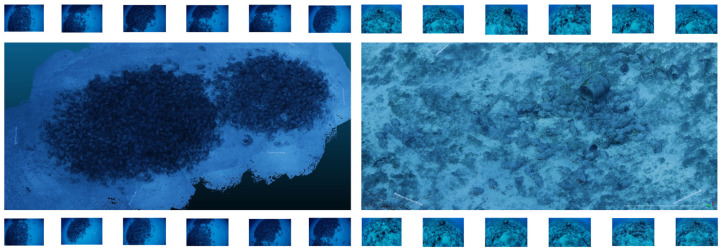
Reconstruction of two different underwater scenes.

The second dataset (dataset B) comprised 200 underwater images collected from a site near the location of the first dataset. This new dataset shared similar depth characteristics with the initial one but featured a large amphora surrounded by several smaller ones. Figure 5 visualizes the dataset, presenting one reconstruction for each dataset along with selected images used in creating the 3D model.

### 4.2. Results

The experiments on dataset A were the first to be conducted. For brevity, the best results from the 22 colormaps are presented in Table 2, showcasing the top-performing ones. For this dataset, the best colormaps were the bone, jet, rainbow, cool, and twilight_shifted. It is important to note that while reconstruction points provide additional information, more points do not necessarily result in better stitching. The non-enhanced images detected an average of 12,094 features per image. In contrast, the enhanced images showed a significant improvement, detecting 13,051 features, an increase of 7.91%. Using the colormap bone, the number of detected features rose to 13,403, representing a 10.82% increase. The OpenSfM methodology processes unprocessed images for 3D reconstruction and reconstructs the 3D model based on them, while OpenSfM-C is an enhanced version that utilizes colormaps to improve feature detection before reconstructing the 3D model from the original images. The specific colormap used in each case is indicated in parentheses.

To evaluate the impact of colormaps on the reconstruction process, it is essential to compare OpenSfM-C with the standard OpenSfM method without colormaps. This direct comparison allows isolation and assesses the contribution of colormaps in enhancing feature detection and overall reconstruction quality. Comparing OpenSfM-C with entirely different reconstruction methods would primarily highlight differences between methodologies rather than isolating the specific effect of colormap usage. To accurately assess how the proposed approach that utilizes colormaps contributes to the reconstruction process, it is essential to compare OpenSfM-C with the standard OpenSfM method without colormaps. This approach ensures that the evaluation focuses on the impact of colormaps rather than broader variations in reconstruction techniques.

Following the initial experiments, dataset B, the second experimental dataset, demonstrated even more pronounced improvements with colormaps utilization compared to dataset A. The initial non-enhanced images in dataset B had an average of 13,297 features per image. Enhancing these images increased the feature count to 14,813, marking an 11.40% improvement. The rainbow colormap achieved the best result, with 21,823 features, representing a remarkable 64.11% increase. In dataset B, most colormaps significantly increased the number of features, highlighting the effectiveness of colormaps in enhancing reconstruction features, particularly in underwater scenarios. The top dataset B colormaps were the jet, summer, rainbow, hsv, and twilight_shifted. Some of them (jet, rainbow, twilight_shifted) were in both datasets, while HSV and summer were at the top only for dataset B. The top results are shown in Table 3.

**Figure 6:**
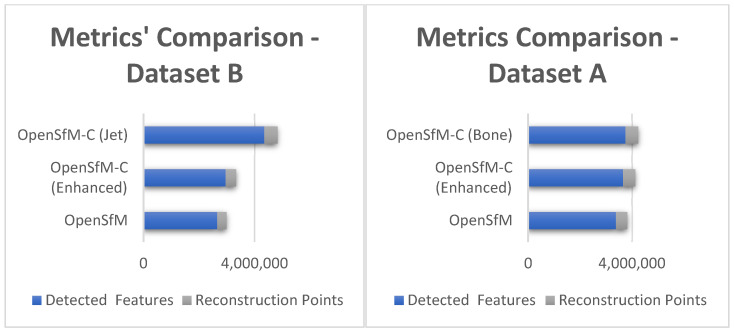
Visual representation of results for datasets A and B.

Each dataset underwent 24 reconstructions, with various implementation factors tested. Several keypoint detection methods were evaluated, with SIFT emerging as the fastest while still detecting numerous keypoints. The colormaps’ utilization often increased the detected keypoints number, with each reconstruction taking approximately an hour for 200–280 input images. Additionally, the reconstruction was performed using the initial images, ensuring that the color differences introduced by the colormaps did not affect the final result. The colormaps assisted solely in the stitching process, leading to a 3D model with better-stitched images and enhanced details. Although colormaps slightly extended the processing time due to the higher number of detected keypoints, this increase was minor compared to the significant match improvement. While colormaps inherently enhance data and contribute to improved feature detection, the results also demonstrate an increase in reconstructed points. This indicates that not only are more features being detected, but these additional features contribute to a denser and more informative point cloud. The increase in reconstructed points suggests that the enhancement introduced by colormaps leads to a more complete and detailed representation of the scene, supporting the claimed contribution. A histogram comparison is provided in Figure 6 to visually illustrate the differences between the results for datasets A and B.

**Figure 7:**
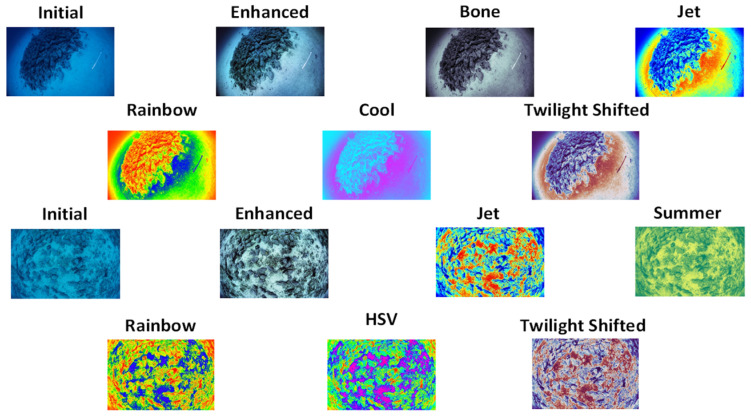
Visualization of the top colormaps for each dataset.

For those seeking to maintain accuracy without extended reconstruction times, sampling techniques can be combined with colormaps to achieve similar results with fewer images. This approach will be explored in future work. In Figure 7, a visualization of different colormaps applied to two distinct datasets is shown. This figure demonstrates how colormaps revealed details that were difficult to see in the original images, enabling SIFT to detect more keypoints than in the initial images. In addition to testing all the colormaps to see if they could enhance the reconstruction process, an approach to identify the best colormap for each dataset was developed.

Several methodologies were explored, but were not integrated in the end. Initially, a method was implemented that involved taking two random continuous images and applying each colormap to them while counting the matches. Although this method quickly identified which colormap might fit best, it relied on only two images and could potentially mislead results since the reconstruction process depends on all dataset images. To address this, a heuristic approach was implemented. This approach first tested all 22 colormaps and then iteratively removed the worst-performing ones until the best colormap was identified. A cumulative results list was maintained to further refine this process, where the match number from each new test was added to the previous results. This method reduced the chances of dismissing a colormap that performed poorly on one image but yielded good results on others.

The heuristic approach results for dataset A are shown in Table 4. Four different experiments were conducted, and each time, a different colormap emerged on top due to the randomness in the selected image pairs from the dataset. The table includes the colormap names that achieved the highest overall scores in terms of matches, with the first row representing the best colormap and the rest following in descending order. It is observed that in the first experiment, which included the top colormaps in the top positions, the total matches were higher than in the other experiments. This indicates that these colormaps achieved the highest results and should be prioritized for testing.

Notably, the jet colormap consistently ranked the second best for reconstruction, while rainbow and twilight_shifted followed in third and fourth place, respectively. These colormaps appeared in every experiment set, indicating that this methodology can reliably recommend suitable colormaps to users. The hsv colormap, which topped three experiments, did not yield good results in reconstruction. The slight inconsistency in identifying the absolute best colormaps for 3D reconstruction arises because the heuristic approach examines only pairs of images. In contrast, the reconstruction process involves each image matching with all others. This limitation is intentional to reduce computational costs while still providing useful colormap recommendations. Interestingly, the jet, rainbow, and twilight_shifted colormaps also performed well for dataset B reconstruction, further validating their effectiveness in underwater environments featuring similar colors.

The results for dataset B were similar to those for dataset A, with jet and rainbow appearing in all cases. Colormaps jet and rainbow frequently occupied the top positions and proved the best colormaps for 3D reconstruction. The colormap twilight_shifted was included in 75% of results and was also a colormap that was very good in reconstruction. Although twilight was also present in dataset A, it did not rank among the top colormaps for reconstruction in dataset B. Interestingly, a different colormap, named turbo, appeared in dataset B but was not among the best in terms of reconstruction.

In dataset B’s case, the heuristic approach worked much better than dataset A, as it identified the top colormaps in all four experiments. It demonstrates that the heuristic approach can effectively provide valuable suggestions about which colormaps to use. The difference in results between datasets A and B highlights the necessity of applying this approach to each dataset individually to account for unique characteristics. Although the best colormaps are often similar across similar datasets, they differ. Therefore, having a methodology that recommends the best colormaps for each dataset can be extremely helpful. The heuristic methodology required approximately 30 min to identify the best colormap when combined with image downscaling, which did not affect the findings. The colormap selection is not a simple task, as it requires performing the 3D reconstruction with all the colormaps to choose the best one. By using this method, the optimal colormap can be found without losing much time. Reconstructing all 22 colormaps in a dataset with 200 images needs approximately 22 h, which shows how important the heuristic approach is, which needed only 30 min to find the best colormap and 1 h for reconstruction. It is also important to note that heuristic results were different from the first iteration’s, which applied 22 colormaps and sorted them according to their matches. The results are shown in Table 5.

The computational costs of the image processing pipeline were evaluated based on key stages, including image enhancement, colormap creation, and reconstruction. All times are for high-resolution images (5760 × 3840). The image enhancement process took approximately 10 min and 18 s for 280 images, averaging 2.21 s per image. The time needed for a heuristic to find the best colormap was 12 min and 40 s. The colormap creation stage demonstrated improved efficiency, processing the same number of images in 2 min and 20 s, achieving a speed of 1.99 images per second. Regarding reconstruction, the colormap-based reconstruction required 1 h and 56 min, while unprocessed reconstruction took slightly longer at 2 h and 1 min, highlighting the computational efficiency associated with colormap processing. These results emphasize the trade-off between processing time and the benefits of enhanced visualization, demonstrating the efficiency of colormap creation while acknowledging the substantial time investment required for high-resolution image enhancement and reconstruction.

In terms of hardware usage, image enhancement utilized 24% of the CPU and 231.6 MB of memory. The heuristic finding process was more computationally demanding, utilizing 75.8% of the CPU while maintaining a memory usage of 229.6 MB. Colormap creation had a lower CPU load at 13.5% but required significantly more memory, reaching 974.7 MB. For 3D reconstruction, the process without colormap used 37.5% of the CPU and 6290.9 MB of memory, whereas reconstruction with colormap had a similar CPU usage of 37% but a higher memory consumption of 7128.9 MB. None of these processes utilized the GPU, indicating that all computations were performed on the CPU.

## 5. Conclusions

This paper introduced a novel method for enhancing 3D reconstruction using colormaps and image enhancement techniques. Additionally, a heuristic methodology was proposed to identify the most effective colormaps for each dataset. This approach provided more image features, leading to better stitching and a more detailed 3D model. The methodology was tested using two real-world datasets, demonstrating significant improvements. It did not add much substantial time to the existing process and proved highly effective. The heuristic methodology showed promising results by consistently identifying the top colormaps. It found three out of the four best colormaps in 100% of the experiments in dataset A. In dataset B, the top two colormaps were identified in all of the experiments, and the third-best colormap was included in the top four best colormaps in half of the experiments. This methodology can work quickly, in less than 30 min, providing users with valuable colormap suggestions.

The results demonstrated that this framework could enhance a state-of-the-art SfM system, leading to more complete and accurate scene reconstructions, particularly in challenging lighting conditions such as underwater environments. The proposed enhancement method resulted in improvements of 7.91% for Dataset A and 11.4% for Dataset B. Additionally, the use of colormaps further increased the number of features per image by 10.82% for dataset A and 64.11% for dataset B. These findings highlight the effectiveness of colormaps, especially in poor lighting and underwater scenarios. The real-world dataset confirmed that this methodology is practical and can significantly improve 3D reconstruction.

One limitation of the method is the potential increase in reconstruction time when utilizing the introduced features. The calculation of matches becomes more challenging due to the higher number of features present in each image, leading to increased complexity in match searching. This issue was addressed by employing sampling techniques to reduce the number of input images and accelerate the matching process, which can improve computational efficiency. In future work, other features aimed at enhancing the accuracy of 3D reconstruction or reducing computational costs to achieve optimal efficiency will be explored and evaluated. This can be achieved using sampling methods together with the proposed method for increasing the matches between images. This approach will allow the results to maintain good detail despite a reduced dataset, thanks to the use of colormaps, and produce results faster due to the sampling.

In future work, the methodology will be extended to cover a wider range of environments. Currently, the focus has been on underwater scenarios, as datasets for such environments are more readily available. However, to further evaluate the generalization capabilities of the method, efforts will be made to incorporate datasets from other challenging environments. These additional scenarios will provide valuable insights into the adaptability of the method to different conditions and help validate its performance in more diverse real-world applications.

## Figures and Tables

**Table 1:** Comparison of the proposed approach with related work.

Paper	Real Conditions	Cost-Effective	Multiple Datasets	Scalability	Effective in Poor Light
[8] Manhattan-world assumption		✓	✓		
[9] Learnable kernels for reconstruction		✓			
[14] Reconstruction of Characters	✓	✓		✓	
[16] Fiducial markers in OpenSfM		✓			
[19] Reconstruction using global context	✓	✓		✓	
[22] Sonar and LiDAR fusion	✓				✓
[30] UAV photogrammetry	✓	✓		✓	
Reconstruction with colormaps	✓	✓	✓	✓	✓

**Table 2:** 3D model reconstruction results for dataset A.

Methodology	Detected Features	Features per Image	Reconstruction Points
OpenSfM	3,386,593	12,094	434,644
OpenSfM-C (enhanced)	3,654,379	13,051	473,649
OpenSfM-C (bone)	3,752,992	13,403	488,235
OpenSfM-C (jet)	3,643,488	13,012	465,777
OpenSfM-C (rainbow)	3,680,873	13,145	468,360
OpenSfM-C (cool)	3,488,480	12,458	447,819
OpenSfM-C (twilight_shifted)	3,613,958	12,906	477,289

**Table 3:** 3D model reconstruction results for dataset B.

Methodology	Detected Features	Features per Image	Reconstruction Points
OpenSfM	2,659,585	13,297	332,970
OpenSfM-C (enhanced)	2,962,642	14,813	377,214
OpenSfM-C (jet)	4,351,002	21,755	477,549
OpenSfM-C (rainbow)	4,364,635	21,823	461,357
OpenSfM-C (summer)	3,330,044	16,650	396,215
OpenSfM-C (hsv)	3,624,432	18,122	377,129
OpenSfM-C (twilight_shifted)	4,143,279	20,716	466,581

**Table 4:** Heuristic colormap selection approach results for dataset A.

1st Experiments		2nd Experiments		3rd Experiments		4th Experiments	
Colormap	Matches	Colormap	Matches	Colormap	Matches	Colormap	Matches
Jet	2,938,449	HSV	1,810,393	HSV	1,676,203	HSV	1,751,360
Rainbow	2,904,357	Jet	1,061,223	Jet	1,241,391	Jet	1,085,490
Twilight_shifted	2,875,798	Rainbow	1,029,413	Rainbow	1,089,825	Rainbow	1,033,242
Twilight	2,412,982	Twilight_shifted	1,009,980	Twilight_shifted	1,062,671	Twilight_shifted	1,014,891

**Table 5:** Heuristic colormap selection approach results for dataset B.

1st Experiments		2nd Experiments		3rd Experiments		4th Experiments	
Colormap	Matches	Colormap	Matches	Colormap	Matches	Colormap	Matches
Jet	3,840,136	Rainbow	3,892,886	Jet	4,002,670	Jet	3,636,218
Twilight_shifted	3,820,115	Twilight	3,588,457	Rainbow	3,747,480	Twilight	3,366,883
Rainbow	3,785,133	Jet	3,361,172	Twilight	3,724,774	Rainbow	3,311,165
Twilight	3,522,048	Turbo	2,501,619	Twilight_shifted	2,674,504	Turbo	2,539,508

## Data Availability

Data is contained within the article.
